# Disparities and Equity Dashboards in the Neonatal Intensive Care Unit: A Qualitative Study of Expert Perspectives

**DOI:** 10.21203/rs.3.rs-3002217/v1

**Published:** 2023-06-26

**Authors:** Sheila Razdan, Laura Hedli, Krista Sigurdson, Jochen Profit, Christine Morton

**Affiliations:** University of British Columbia; Stanford University

## Abstract

**Objective:**

Racial/ethnic disparities are well-described in the neonatal intensive care unit (NICU). We explore expert opinion on their root causes, potential solutions, and the ability of health equity dashboards to meaningfully address NICU disparities.

**Study Design:**

We conducted 12 qualitative semi-structured interviews, purposively selecting a diverse group of neonatal experts. We used grounded theory to develop codes, shape interviews, and conduct analysis.

**Result:**

Participants identified three sources of disparity: interpersonal bias, care process barriers, and social determinants of health, particularly as they affect parental engagement in the NICU. Proposed solutions included racial/cultural concordance, bolstering hospital-based resources, and policy interventions. Health equity dashboards were viewed as useful but limited because clinical metrics do not account for many of the aforementioned sources of disparities.

**Conclusion:**

Equity dashboards serve as a motivational starting point for quality improvement; future iterations may require novel, qualitative data sources to identify underlying etiologies of NICU disparities.

## Introduction

For mothers and infants, significant disparities in health care and outcomes persists across the pregnancy, birth and early childhood continuum (1, 2). Black women are nearly twice as likely to experience a preterm birth (3) and are more than twice as likely to die in the peri- and post-partum period compared to White women (4). Interpersonal racism experienced by Black mothers has been theorized as an independent risk factor for preterm delivery (5), and qualitative research demonstrates that Black mothers experience racism while their children are treated in the NICU (6). Neglectful care, judgmental care, and systemic barriers may lead to suboptimal care in the NICU, while priority treatment can may lead to privileged care (7). A systematic review demonstrated that Black and Hispanic infants are more likely to be born in lower quality hospitals, in addition to receiving differential care within the NICU (8). Like other forms of healthcare experienced in pregnancy and birth, the NICU does not provide a protective social cocoon from racial inequities.

Quality improvement (QI) methods are one approach suggested to achieve health equity, including using quality of care measures that are family-centered and incorporate NICU families’ voices (9, 10, 11, 12, 13). Obtaining data is critical to sustained improvement work; without stratification of quality metrics by race/ethnicity, or other attributes that may confer therapeutic disadvantage, differential care may remain hidden. Perinatal quality collaboratives can utilize dashboards to identify disparities to motivate improvement efforts (14, 15).

**Figure 1.**
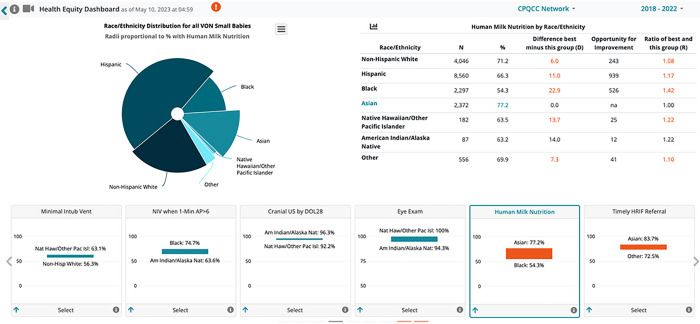
Sample of California Perinatal Quality Care Collaborative Health Equity Dashboard

Dashboards are tools used in many areas of healthcare to disseminate data to a wide audience and inform policy change (16), such as highlighting racial disparities throughout the Covid-19 pandemic (17), visualizing social determinants of health (18, 19), and tracking global equity efforts in vaccination (20). To promote equity-based QI, the California Perinatal Quality Care Collaborative (CPQCC) developed a novel health equity dashboard for neonatal intensive care units (NICUs), which includes unit-specific, race/ethnicity-stratified data on NICU processes (e.g., breast milk use at discharge) and outcomes (e.g., chronic lung disease) (Fig. 1). Each unit can compare its overall and stratified metrics to the CPQCC average, identifying unit-specific strengths as well as areas for improvement. However, at this stage, these metrics do not necessarily show the sources of disparities in NICU quality of care or their potential solutions.

Our aims in this qualitative interview study were to understand perceived sources of disparities in NICU quality of care and outcomes, explore potential solutions, and understand how health equity dashboards can meaningfully capture this information.

## Subjects and Methods

### Study Design

We conducted qualitative semi-structured interviews from 2016–2017 with 12 experts in neonatal health who were purposively selected based on their area of expertise and to achieve gender, race/ethnicity, and role diversity. Participants identified as men and women, Black, White, Asian, and Hispanic/Latino; they represented neonatologists, nurses, neonatal nurse practitioners, health services researchers, and parents of former NICU patients who advised and advocated for other NICU parents. Because we promised participants anonymity and confidentiality, we do not note their names or individual characteristics. We obtained informed consent and study approval by the Stanford Institutional Review Board.

### Data Collection

We conducted 12 in-depth, in-person or phone interviews using a semi-structured interview guide and adapted interviews depending on the participant’s area of expertise and on topics discussed. The interview guide was designed to solicit experiences with neonatal racial/ethnic disparities, perspectives on what causes disparities and how to eliminate them, particularly with reference to the use of a health equity dashboard. Interviews were audio-recorded and transcribed verbatim prior to analysis.

### Analysis

We analyzed data using Dedoose (Version 9.0.62, Socio-Cultural Research Consultants, LLC), a mixed- methods software platform. We used grounded theory, an inductive and iterative method, to develop codes, shape subsequent interviews, and conduct analysis (21). This method allows themes to be identified in the data, rather than approaching the interviews with predetermined hypotheses. Each transcript was reviewed by at least two investigators with backgrounds in neonatology, sociology, and journalism. Three analysts conferred and arrived at consensus on coding strategies, identifying themes, and data cohesion during regular data analysis sessions.

### Results

**Figure 2.**
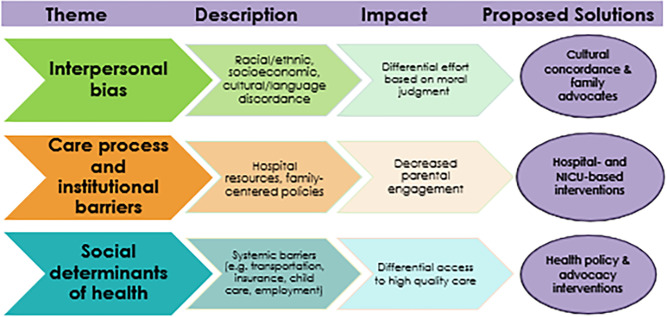
Etiologies of disparities in the NICU. Interviews described interpersonal bias, care process barriers, and social determinants of health, each with its own negative impacts and possible solutions.

We identified three main sources of disparity in NICU quality of care in our participants’ narratives: clinicians’ interpersonal biases, care process barriers, and structural racism, particularly as they affect parental engagement in the NICU (Fig. 2). In our analysis, we demonstrate how these sources of disparity affect care quality. Lastly, we discuss participants’ proposals to achieve equity, including use of data dashboards.

### Interpersonal Bias

When asked about sources of disparities in the NICU, all participants shared specific instances of communications and behaviors that reflect healthcare clinicians’ judgment and treatment of infants and families in the NICU. One source of disparity stems from the bias, either implicit or explicit, that surfaces when NICU clinicians unfavorably judge families who are not frequently at the infant’s bedside, ascribing this absence to parental lack of commitment to the baby’s health journey, rather than intractable circumstances. Healthcare clinicians’ biases around perceived ‘noncooperation’ of parents can result in nurses feeling *“sympathy for the baby, but apathy for the parents who don’t appear to cooperate. They would still care for the baby, but they feel very frustrated with how some parents don’t come on board with the breastmilk pumping, or visiting frequently, or [being] willing to learn.”* The stories we heard about how staff biases can impact quality of care also emphasized the need for a shift in provider attitudes from presuming moral failings of NICU parents who cannot visit their babies often, to understanding that limited parental presence at bedside may reflect concrete constraints on a family’s resources related to transportation, sibling care, employment demands, or other structural factors. One participant related the case of a Latina parent whose infant died and who complained that the nurses had not spent as much time interacting with their family as they did with others, and implied that the reason was their low socioeconomic status. The hospital staff subsequently confirmed from video footage that confirmed that clinicians had entered the room less frequently and spent less time with that infant. While the reasons for this family receiving less attention deserve further exploration, interpersonal biases in the NICU may convey clinicians’ moral judgments to families via their actions and words, and could result in poorer quality of care.

### Priority care goes to the “sweet family” and the “engaged parent”

Interpersonal biases can also move in a positive direction. Participants recounted examples of clinicians favoring parents who they saw often and judged to be more loving than parents who spent little time at the bedside. One example was how nurses rallied around and identified resources for refugee parents who fled ISIS and were now in the U.S. with a preterm infant. Neither parent spoke English, yet the participant noted *“it happened to be an extremely sweet family that we feel like we really want to be sympathetic, we find out as much about them as possible to see if we can help.”* Favoritism or positive interpersonal bias was seen by participants as often based on a family’s “story” and whether they are perceived as deserving.

Not all parents have equal opportunities to be engaged at levels deemed appropriate or optimal by healthcare clinicians. *“Having families there facilitates a relationship being formed with the care providers and the parents, which ultimately helps everybody, including the kid.”* Parents who become engaged—by being intimately involved in their own infants’ NICU experiences, visiting frequently, interacting with the staff, and asking for what they needed—often continue as de facto or official parent advocates or family advisory board members. This trajectory describes the journey of the parent advocates we interviewed. They told us that parent advocates are typically well-resourced and as a result, are able to volunteer their time and expertise. Overall, our participants believed that class and race differences among parents can result in interpersonal biases or differential ability to support NICU families.

### Care Processes and Institutional Barriers

Participants described how unit and hospital policies can reinforce biases. Care processes in the NICU may benefit specific populations, including mothers who speak English and can be frequently present in the NICU during daytime hours. Hospitals may not have sufficient resources or funding to support families with transportation and housing limitations, which can compound bias against parents who aren’t able to be present at the bedside. What follows is not a comprehensive description of institutional-level barriers, but rather a sample from our interviews.

#### Where bias intersects with workflows and care processes

An example of the interplay between bias and unit workflows involves nurse staffing assignments. Participants told us about how families who prefer languages other than English require extra time for clinicians to coordinate translation services and communicate, yet staffing assignments do not account for this additional workload. Our participants recounted numerous examples of what one called *“disparity of attention,”* leading to staff burnout and less time with families when interpreters are not immediately available, audio/video translation services are inoperable, and language barriers seem insurmountable.

Care processes may be different depending on the time of day, leading to another source of disparity that may have differential effects based on the race/ethnicity of parents. We heard that night shift staff may be less open to engaging parents in kangaroo care: *“we have another mom that maybe only comes in one or two nights a week and wants to do kangaroo care, but maybe the night shift nurse is not as willing as the day shift nurse to do it. But that mom can only come in at night because she has two other kids she has to take care of, and she needs to pay her rent and can’t miss a day from work.”* Thus, care favors parents with the flexibility of being present during the day, who are often parents with access to parental leave or a flexible workplace.

### Forgetting about the father

Care process disparities may impact parents differently. While mothers are specifically invited to participate in breastfeeding and pumping, fathers may not be given a defined role in caring for their infant. As a result, fathers may get the message that there is no place for them in the NICU. A parent advocate observed that Black fathers often say *“they do not feel welcome at all. And they feel that when they walk in there, all eyes are on them.”* Other study participants did not mention fathers’ involvement in NICU care processes, further emphasizing this potential blind spot and opportunity for improvement.

### Limited housing and transportation resources

Nearly all participants discussed the plight of NICU families who have recently immigrated to the United States or live far from the hospital. While some hospitals now employ private NICU rooms with sleeping accommodations for family members, and others are affiliated with charitable organizations that offer long-term housing for patients’ families, our interviews showed that many families face barriers to bedside presence.

Prohibitive costs impeding parental presence at the bedside may be addressed through public or private funds. One participant noted that at one hospital, high parking fees at a private facility made visiting the NICU cost-prohibitive for some families. One wealthy former NICU parent fundraised to cover all parking fees for all NICU families and also provide public transportation to families who didn’t own cars. *“It has made a huge difference,”* the clinician remarked. *“The biggest difference is actually probably made for your average middle-class family,”* due to the reduced financial burden. Several of the care process and institutional barriers described by our participants underscore the fact that U.S. policies value individualism and lack broad social supports, such as paid family leave, safe and affordable childcare, or reliable, inexpensive public transportation.

### Social Determinants of Health (SDOH)

SDOH, or structural and social factors that influence health outcomes, were frequently invoked by participants as sources of disparities in the NICU.

### Access to high quality care

Because children’s hospitals operate within highly regionalized systems, SDOH may influence patterns of referrals and access to quality of care. One participant noted how NICU admissions are affected by insurance type and a predetermined referral system, which have the effect of stratifying access by quality: *“To me, that is, in many ways, the most vulnerable part of NICU care and it’s also, I think, the least well-studied…When you see [social] stratification in where the regionalized systems are sending kids, sooner or later, you’re going to see difference in the quality of services.”* Essentially, families may not have the same access to high-quality health care based on the quality of their health insurance plans: *“If you believed NICUs have efficacy, which all the evidence suggest, and that’s very powerful efficacy, then all the policies that facilitate equitable referral need to be protected.”* Thus, this differential access to high-quality care is, according to this participant, a major driver of disparities.

### Screening without solutions

Interviewees said healthcare providers often recognize SDOH challenges exist and may even screen for them; however, they may lack the means to improve health equity in their NICU. One participant posited: “*What’s the point of screening about food insecurity, and then people say, ‘Yes, I am food insecure.’ And then you’re, like, ‘Oh, that’s terrible.’…There’s got to be some resource on the other side of it.”* This participant suggested that using a SDOH screener for food insecurity would be unethical without resources that could directly address the issue. Furthermore, even if resources within the NICU exist, another participant emphasized that *“people feel like they can’t do much about [it],”* and therefore SDOH may go unaddressed. This respondent further explained that *“if we don’t think about those social determinants of health, those social factors, then we’re still going to miss the boat in terms of eliminating neonatal health disparities.”* This quote illustrates a common theme among participants that achieving equitable NICU outcomes requires addressing SDOH.

### Potential Solutions to Reducing Neonatal Disparities

Study participants noted many possible, often overlapping, solutions to reducing interpersonal bias, care processes and institutional barriers, and addressing SDOH that drive NICU disparities.

### Improving relationships between families and clinicians

Improving trust between families and clinicians was a potential goal for disparities in the NICU that stem from interpersonal biases. Several participants acknowledged that they did not physically resemble or experientially relate (in terms of race, socioeconomic status, preferred language, or medical experiences) to many NICU families, so the proposed strategy was to increase diversity of healthcare staff.

Another strategy discussed by participants for strengthening trust was devoting more time to patients and families who have historically felt marginalized and mistreated by larger systems: *“With the same problem, I can tell something to the White family and something to the Black family. But the White family trusts the system, so they’re okay. But the Black family, they don’t trust the system so much because they’re aware of what happened in Tuskegee…so they may not trust you. So, you have to spend more time.”* Evoking a racist study from the 1970s, where researchers withheld lifesaving treatment from Black men suffering from syphilis to study its effects, this participant underscored the longstanding legacies of mistrust in healthcare institutions among Black communities. One participant suggested that NICUs should solicit feedback from families who had negative experiences or were not regularly present during their child’s NICU course, to fully understand families’ care experiences.

Another strategy raised for bridging trust issues centers on the role of parent advocates: *“So many of these doctors have children. But yet, they can’t connect with the NICU experience,” said one advocate. “They can think, ‘Oh, yes, having a baby in the NICU must be terrible.’ But until you go through it, you really don’t have the grasp of it.”* Experiential concordance via parent advocates can help families process the impact of having a baby in the NICU and mitigate interpersonal bias through advocacy and bridging communications with clinicians. Obtaining funding and increasing the presence of parent advocates are important administrative considerations.

### Focus on the whole family unit

Future efforts to reduce disparities should incorporate ways to improve the quality of care for the family rather than solely focusing on the infant. One parent participant developed a worksheet and emotional well-being questionnaire for families, which asks basic questions like: While you’re in the NICU, who pays the bills? Washes the dishes? It asks families to provide the names and numbers of those who can assist them. The emotional well-being questionnaire asks about families’ broader goals, including: Where do you see yourself in five years? Such questions may help keep families on track with their everyday responsibilities and also illustrate that their reality in the NICU is only temporary.

### Create policies to reduce care process barriers and increase parental time at bedside

Participants noted that solutions should address unit-based and hospital-wide policies affecting NICU families. For example, acquiring long-term funding to offset the cost of local housing and hospital parking will support families spending more time at bedside. Another solution mentioned was to expand the social work team. Strengthening this workforce would help ensure families are screened for SDOH and directed to appropriate resources. One unique solution related to SDOH access was to leverage information technology platforms to more efficiently link families with social services. In using the platform, explained the participant: *“If I come to the NICU and tell you that I’m hungry, you can tell me, as a NICU provider, what agencies might be available in my community to help me so that I can find something to eat.”* Another proposed intervention was improving access to interpreter services, accounting for the additional time it takes to communicate with families who have limited English proficiency, and adjusting staffing accordingly. Overall, participants emphasized the importance of strengthening multiple institutional policies, resources, and workflows, rather than taking a one-size-fits-all approach. As a clinician shared with us: *“I think we have to realize that families are going to need different things in order just to have equal opportunity.”*

### Focus upstream of the NICU

Many participants felt that the key to improving disparities in the NICU was to first tackle the racism that mothers of color experience. As a participant explained: *“I think where you want to intervene is some place upstream. So, either help women not have to experience racial discrimination or give them coping skills to handle it when it occurs.”* In this example, the participant shows that neonatal outcomes are inseparable from maternal experience. Reducing the racism and the impact of racism that women of color experience is critical to improving maternal health, and subsequently infant health.

### Advocate for improved health policy

Many participants acknowledged the impact of SDOH and the need for upstream interventions via health policy and advocacy, though most did not have specific interventions in mind. Specifically, one clinician noted that funding for social services in the U.S. is lacking when compared to other high-resource nations, and as a country, the U.S. should consider increased funding for safety net policies and addressing differences in SDOH.

### The utility of a health equity dashboard

In addition to novel solutions proposed by participants, we asked about the utility of a health equity dashboard in reducing disparities. Many participants noted real-time data would significantly influence unit-specific practices as well as statewide and nationwide policy. Three clinicians believed an equity dashboard could motivate staff in the unit to want to improve, especially if it identifies *“low-hanging fruit.”* Another participant suggested dashboard displays could highlight both positive outcomes and metrics that could use improvement. As these participants note, the presence of positive data could help inspire more action for the outcomes where disparities are reported.

Some participants felt a dashboard would address inequities that have often gone unmeasured. A parent advocate noted that the dashboard would help units proactively confront disparities rather than relying on observation to guide changes. More specifically, one clinician felt that an equity dashboard would allow them to better understand outcomes among the growing Asian American population in their own community, who are not typically included in research studies. They explained, *“I don’t want to do a bad job counseling [on outcomes] based on network studies that have looked at primarily indigent Black, White, Hispanic babies…it would give me a bit more direction.”* By including statewide data, the dashboard could allow providers to have more informed conversations with NICU families. Another clinician noted this dashboard could inspire *“every hospital…to have a committee to address this issue [of disparities]…something that’s doable, something that can be replicated,”* which could then be translated into unit or hospital policy.

Another theme we identified was the importance participants placed on putting dashboard data into context. As one participant noted, *“Just putting things out for the staff without an explanation or going through it has not been helpful…they just glaze over it.”* Instead, one participant suggested that the dashboard should provide a user manual that provides guidance for how to interpret the metrics. Another clinician noted the dashboard should both *“help them understand why they’re doing badly and ways that they could potentially do better,”* drawing on examples of what others have successfully done rather than reinventing the wheel. Highlighting positive deviants, or top performers, may serve as a more powerful motivator than emphasizing units with poor outcomes. One clinician said that the examples and solutions provided by the dashboard should be realistic for the unit using it, because *“some of the staffing needs are unfortunately not something the hospital can change easily day by day.”* Unit demographics, such as the average census or staffing ratios, could contextualize results.

Moreover, participants noted that many of the etiologies of disparities, especially those related to interpersonal bias and furthering family-centered care, may not be captured in current equity dashboards. One parent advocate wondered if a dashboard is able to fully reflect the NICU experience. They questioned, *“How well are we doing in caring for the family, meeting their needs to the best of their ability, really furthering family centered care for them, giving them the most and best resources that we can to have them continue on with a healthy baby at home?”* To address these concerns, ideas for future directions for the dashboard included capturing data through a survey designed by families for families. This survey would include whether families were mentored by a parent advocate and who in the NICU provided emotional support.

## Discussion

This study aimed to identify the etiology of NICU disparities through interviewing a diverse group of experts and to understand the how a health equity dashboard could contribute toward reducing disparities in NICU quality of care. As a clinician observed, *“for an outsider looking in, you would think all babies would have more of an equal chance.”* Indeed, a common refrain from NICU clinicians is: *“we treat all babies the same here.”* Yet, disparities in care continue to exist, as evidenced by our participants’ accounts and previous literature (7). Our participants explicitly called out interpersonal bias, institutional barriers, and differences in SDOH but often only hinted at an underlying contributor: structural racism. In our study, we identified interpersonal racism in the form of clinician bias, institutional racism as it affects care process barriers, and structural racism manifesting via inequities in SDOH and care referral patterns (6). Our analysis drew out how these three levels of racism contribute to differential access to the NICU and parental involvement at the bedside. Participants conveyed that the etiology of disparities is multifactorial with racism being one of many contributing factors analyzed here. This disparity in engagement may affect families’ trust in the care team (22), processes and outcomes in the NICU, such as developmental care (23, 24, 25), and even long-term follow up after discharge (26).

Participants grappled with how to address disparities, and how a dashboard can capture the metrics needed to make meaningful change toward health equity. The current version of the health equity dashboard displays unit-specific process and outcome metrics that can be stratified by race/ethnicity and compared to other units. However, it does not contain any family-centered care measures, such as time spent at bedside or holding. Family-centered care has been emphasized as an important domain in NICU quality improvement (27, 28), and family-centered care initiatives have been shown to decrease stress and improve confidence in NICU families (29). One suggestion was to increase the presence of family advocates, particularly those who come from diverse backgrounds, who are typically underrepresented on hospital family councils (30). Future dashboard iterations could try to capture family-centered care metrics as well as proxies of institutional and structural racism, such as neighborhood social and built environment characteristics (31, 32, 33).

While we interviewed a limited number of participants, our study draws from a diverse group in terms of gender, race/ethnicity, profession, and geographic location of practice; importantly, it also includes parent advocates, following recommendations from recent NICU health equity literature (34, 35). Thus, it builds on previous qualitative and mixed method studies that examine examples of and prevalence of bias in the NICU (7, 36). A limitation of the study was that the health equity dashboard was not officially released until after the interviews, so participants were commenting on a theoretical model of the dashboard. As a result of this study, there are efforts to update the dashboard to include family-centered care measures, add stratification by language preference, add longitudinal trend analyses, link metrics to neighborhood attributes, and eventually develop a library of community resources and other potential solutions.

Our findings suggest that while health equity dashboards are useful to uncover disparities in the NICU, they may be limited in measuring the interpersonal, institutional, and structural racism that families experience, though they may serve as a motivational starting point for the quality improvement journey. Future research should focus on evaluating their usability, feasibility, and impact in motivating equity action.
